# Passive Surveillance for Azole-Resistant *Aspergillus fumigatus*, United States, 2011–2013

**DOI:** 10.3201/eid2009.140142

**Published:** 2014-09

**Authors:** Cau D. Pham, Errol Reiss, Ferry Hagen, Jacques F. Meis, Shawn R. Lockhart

**Affiliations:** Centers for Disease Control and Prevention, Atlanta, Georgia, USA (C.D. Pham, E. Reiss, S.R. Lockhart); Canisius Wilhelmina Hospital, Nijmegen, the Netherlands (F. Hagen, J.F. Meis);; Radboud University Medical Centre, Nijmegen (F. Hagen, J.F. Meis)

**Keywords:** antifungal resistance, azole, azole resistance, fungi, Aspergillus fumigatus, CYP51A, TR34/L98H, susceptibility testing, passive surveillance, United States

## Abstract

Emergence of *Aspergillus fumigatus* strains containing mutations that lead to azole resistance has become a serious public health threat in many countries. Nucleotide polymorphisms leading to amino acid substitutions in the lanosterol demethylase gene (*cyp51A*) are associated with reduced susceptibility to azole drugs. The most widely recognized mutation is a lysine to histidine substitution at aa 98 (L98H) and a duplication of the untranscribed promoter region, together known as TR_34_/L98H. This mechanism of resistance has been reported in Europe, Asia, and the Middle East, and is associated with resistance to all azole drugs and subsequent treatment failures. To determine whether isolates with this mutation are spreading into the United States, we conducted a passive surveillance–based study of 1,026 clinical isolates of *A. fumigatus* from 22 US states during 2011–2013. No isolates harboring the TR_34_/L98H mutation were detected, and MICs of itraconazole were generally low.

## Abstract 2

*A*. *fumigatus cyp51A*–mediated resistance to azole drugs is rare in the United States.

Azole antifungal drugs are the first line of therapy against *Aspergillus fumigatus*, a common etiologic agent of aspergillosis. These drugs are used as empirical prophylaxis and as targeted therapy for invasive aspergillosis. Resistance to azole drugs has been associated with treatment failure and deaths in patients with aspergillosis. In the past 2 decades, azole resistance in *A. fumigatus* has been documented in many regions (*1**–**7*). The high prevalence of azole resistance in *A. fumigatus* has prompted the European Centre for Disease Prevention and Control to increase the risk level of this organism to a public health risk (*8*).

A common mechanism that confers resistance to azole drugs is a mutation in the lanosterol 14 α-demethylase gene that encodes the CYP51A protein. This protein is the primary target of azole drugs and sterol demethylation inhibitor (DMI) fungicides (*9**–**11*). However, not all *cyp51A* mutations contribute to azole resistance (some are benign), and not all azole resistance is caused by mutations in the *cyp51A* gene (*12*).

In the past decade, novel *cyp51A* promoter duplication mutations, especially the cooperative pair of *cyp51A* mutations known as TR_34_/L98H, have emerged as a predominant azole-resistance mechanism in *A. fumigatus* (*1**,**4**,**6**,**10**,**11**,**13**–**22*). Isolates harboring the TR_34_/L98H mutation are cross-resistant to multiple azoles (*4**,**15**,**17*). A second *cyp51A*-promoter duplication genotype, the recently discovered TR_46_/Y121F+T289A, also displays high tolerance for voriconazole (*13**,**14*). Both genotypes are associated with in vitro MICs that exceed by multiple dilutions the established epidemiologic cutoff value (ECV) of ≤1 μg/mL published by the European Committee on Antimicrobial Susceptibility Testing and Clinical and Laboratory Standards Institute members for medical azole agents (*9**,**13*). Moreover, these mutations often contribute to failure of azole therapy in aspergillosis patients (*13**–**23*). Their presence rules out the use of voriconazole, the treatment recommended by the Infectious Diseases Society of America for aspergillosis, and leaves amphotericin B, an antifungal drug with toxic side effects, as the primary therapy choice.

Since the discovery of the TR_34_/L98H mutation in the Netherlands by Verweij et al. in 2007 (*15*), other countries in Europe, including Austria, Belgium, Germany, Spain, France, the United Kingdom, Denmark, and Norway, have also reported isolates harboring TR_34_/L98H (*5**,**7**,**16**–**20**,**24*). *A. fumigatus* isolates with the TR_34_/L98H mutation have also been reported in China, India, and Iran (*1**,**4**,**6**,**21*). Because of rapid increase in detection of the TR_34_/L98H mutation in many regions and its adverse effect on patient management, isolates harboring this mutation pose a serious public health threat. Isolates bearing the TR_34_/L98H mutation have not been documented in United States, but given the number of aspergillosis cases in this country, its presence could pose a serious public health threat, as it does in Europe.

The TR_34_/L98H mutation is not associated with use of long-term medical azole therapy in patients. Instead, prolonged exposure of the fungus to sterol DMI fungicides in the environment, in conjunction with sexual reproduction, has probably led to dissemination of this mutation (*19**,**25*). As in many countries in which this genotype has been reported, DMI fungicides are used in various agricultural practices across the United States. According to the US Department of Agriculture data for 2010–2013, DMI fungicides, such as tebuconazole, propiconazole, prothioconazole, tetraconazole, metconazole, and epoxiconazole, have been used by several agricultural producers (*26*). This shared agriculture practice indicates that the TR_34_/L98H mutation could potentially arise in the United States.

Thus, the Centers for Disease Control and Prevention initiated passive surveillance for *A. fumigatus* isolates in 2011 to identify resistance to the representative azole itraconazole. More than 1,000 clinical isolates from 22 states were screened by using an itraconazole antifungal plate assay and the Etest to measure itraconazole MICs. For isolates that require an MIC greater than the ECV, DNA sequence analysis of the *cyp51A* gene did not yield any isolates with the TR_34_/L98H genotype.

## Materials and Methods

### Fungal Isolates

*A. fumigatus* isolates were obtained through the American Society for Microbiology listservs (DivC) and (ClinMicroNet) (http://www.asm.org/index.php/online-community-groups/listservs) and through the US Association of Public Health Laboratories. The request for isolates read as follows: “To determine the US level of *A. fumigatus* azole resistance, we are requesting submission of: 1) All new or stored isolates of *Aspergillus fumigatus* from US collections; 2) Any new isolates of *A. fumigatus* regardless of clinical relevance.” Isolates were sent to the Centers for Disease Control and Prevention where the species was confirmed by colony color and morphologic features observed by microscopy. Cryptic species that required increased MICs were subsequently ruled out by DNA sequencing of the *cyp51A* gene as described below.

### Itraconazole Susceptibility Assay

All isolates were screened by using 2 methods: antifungal plate culture and itraconazole Etest (bioMérieux, Marcy l’Etoile, France). For plate culture, a 24-well culture plate containing 1 mL of Sabouraud agar supplemented with 4 μg/mL of itraconazole was used. Twenty microliters of a suspension containing ≈ 2 × 10^4^ conidia were transferred to each well and allowed to dry at ambient temperature for 30 min before incubation. Etest determination of itraconazole MICs was performed as described by Pfaller et al. (*27*) with minor modifications. Isolates of *A. fumigatus* were allowed to sporulate on potato dextrose agar slants for 5–7 days. Conidial suspensions were prepared in sterile distilled water containing 1% Tween-20. The concentration was adjusted to an optical density of 0.09–0.13 or ≈10^6^ conidia/mL equivalent at 530 nm. An RPMI 1640 agar plate was inoculated by streaking a conidia-laden cotton swab bidirectionally across the surface of the plate. An itraconazole Etest strip was placed across the central surface of the freshly inoculated RPMI plate. An azole-resistant and an azole-susceptible isolate for which broth microdilution azole MICs were known were used as controls (*1*). The Etest and itraconazole plates were incubated at 37°C, and results were recorded 48 hours postinoculation.

### Genetic Profiling of c*yp51A*

DNA sequence analysis of the *cyp51A* gene and its promoter region was performed for all isolates that required an MIC above the ECV (1 μg/mL) by using primers described (*1*). A subset of the first 561 isolates was also examined by using a mixed-format real-time PCR to detect nucleotide polymorphisms that cause single amino acid substitutions at Gly54, Leu98, Gly138, and Met220, and the recently discovered TR_46_/Y121F/T289A in the CYP51A protein as described by Klaassen et al. (*28*).

*A. fumigatus* genomic DNA was purified by using the DNeasy blood and tissue kit (QIAGEN, Valencia, CA, USA) with some modifications. Hyphae were transferred to a 1.5-mL microcentrifuge tube containing 360 μL of Z-buffer (*29*) (60 mmol/L Na_2_HPO_4_, 40 mmol/L NaH_2_PO_4_, 10 mmol/L KCl, 1 mmol/L MgSO_4_, 38 mmol/L β-mercaptoethanol, pH 7) and 40 μL of proteinase K (QIAGEN). A total of 250 mg of 0.5-mm zirconia beads (BioSpec, Bartlesville, OK, USA) was added to the cell suspension, and cells were homogenized for 1 min at maximum speed by using a mini-beadbeater (BioSpec). Four hundred microliters of AL buffer (QIAGEN) was added to the cell lysate and mixed by vortexing for 10 s. The lysate was then centrifuged at 13,200 rpm for 5 min. The supernatant was transferred to a clean 1.5-mL microcentrifuge tube, and an equal volume of absolute ethanol was added to the supernatant. After briefly undergoing vortexing, the DNA suspension was transferred to a spin column (QIAGEN). DNA binding, washes, and elution steps were followed as described by the manufacturer.

## Results

### Characteristics of *A. fumigatus* Isolates

**Figure 1:**
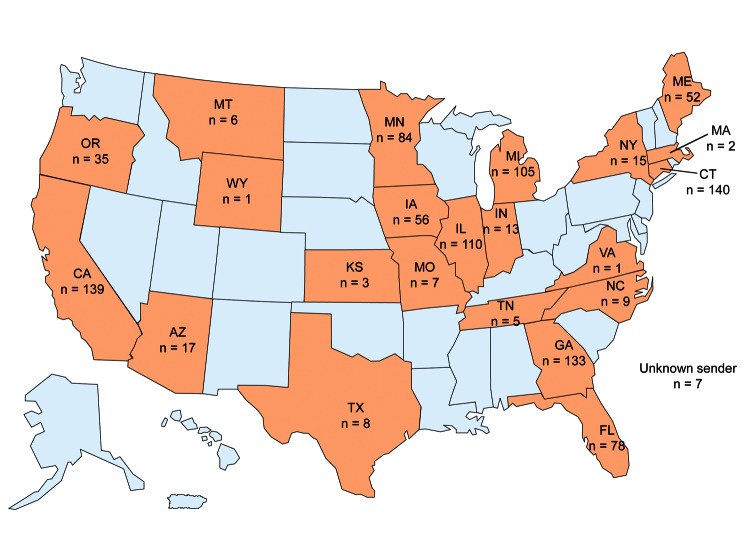
Distribution of *Aspergillus fumigatus* isolates, United States, 2011–2013. A total of 1,026 clinical isolates were received from 22 states during October 2011–October 2013.

A request for *A. fumigatus* clinical isolates collected beginning in 2011 was initiated in June 2011. Because the TR_34_/L98H mutation was believed to be environmentally induced, all *A. fumigatus* clinical isolates were accepted, not just those causing infection. A total of 1,026 clinical isolates were obtained during September 2011–September 2013 from hospitals, clinics, and state public health laboratories across 22 states (Figure 1), including Arizona (n = 17), California (n = 139), Connecticut (n = 140), Florida (n = 78), Georgia (n = 133), Iowa (n = 56), Illinois (n = 111), Indiana (n = 13), Kansas (n = 3), Massachusetts (n = 2), Maine (n = 52), Michigan (n = 105), Minnesota (n = 84), Missouri (n = 7), Montana (n = 6), North Carolina (n = 9), New York (n = 15), Oregon (n = 35), Tennessee (n = 5), Texas (n = 8), Virginia (n = 1), Wyoming (n = 1), and unknown (n = 7). The isolates were form respiratory tracts (≈57%), ears (≈4%), other tissues (≈5%), and unknown sources (≈34%). Of respiratory tract isolates, 65% were collected from sputum and 25% from bronchoalveolar lavage specimens.

**Figure 2:**
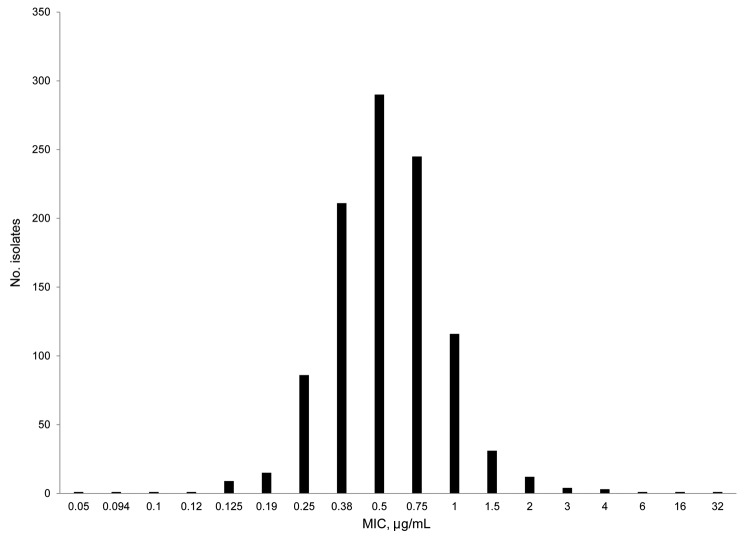
Itraconazole susceptibility profile for *Aspergillus fumigatus* isolates, United States, 2011–2013. The MIC (μg/mL) required by each isolate was determined by using the Etest method. Approximately 5% of the isolates require an MIC higher than the established epidemiologic cutoff value of 1 μg/mL.

Most *A. fumigatus* isolates were susceptible to itraconazole. Historically, the TR_34_/L98H mutation has been detected by itraconazole plate assay. We also used the Etest validate its utility for detecting high MICs. The same 3 isolates were identified by their growth on the itraconazole plate and an itraconazole Etest MIC >4 μg/mL, which indicated congruence for the 2 tests. Overall MICs ranged from 0.05 μg/mL to 32 μg/mL, and 95% of the isolates required an MIC ≤1 μg/mL) (Figure 2). The 50% MIC (MIC_50_,), MIC_90_, and MIC mode for isolates were 0.5 μg/mL, 1.0 μg/mL, and 0.5 μg/mL, respectively. Of 51 isolates that required an increased MIC for itraconazole, >94% (n = 48) required an MIC <4 μg/mL and were negative by agar screening, and 3 isolates required MICs of 6 μg/mL (n = 1), 16 μg/mL (n = 1), or ≥32 μg/mL (n = 1), the maximum value on the Etest strip.

### Genetic Analysis of the *cyp51A* Gene

DNA sequence analysis of the *cyp51A* gene was performed for 51 isolates that required an itraconazole MIC >1 μg/mL and for 36 isolates that required an itraconazole MIC ≤1 μg/mL. The duplication mutations (TR_34_ and TR_46_) in the promoter region of *cyp51A* and the L98H or Y121F/T289A substitutions in *cyp*51A were not detected. Eighteen (35%) of the 51 isolates that required an increased MIC had a *cyp51A* mutation. The most common mutation in this group was a novel valine→isoleucine substitution at residue 242 (I242V), which was found in 13 of the isolates that required increased MICs and 2 of the control isolates; all required MICs ≤4 μg/mL. There was also a set of 4 linked mutations in 2 of the control isolates that are commonly seen in susceptible isolates (*1**,**3*). The only mutation found that has been associated with azole treatment failure was an M220I mutation in the isolate that required an MIC ≥32 μg/mL. A mixed-format real-time PCR was also used to screen the first 561 isolates collected for other known resistance-inducing *cyp51A* single nucleotide polymorphisms. Except for the M220I mutation, no other polymorphisms were identified by using this method.

## Discussion

Azoles, specifically voriconazole and (in some instances) itraconazole, are the recommended treatment for aspergillosis (*30*). These drugs have a high potency against the most common causes of aspergillosis (i.e., *A. fumigatus*, *A. niger*, *A. flavus*, *and A. terreus*). However, reduced susceptibility to these drugs has been reported (*3**,**7**,**15**,**18*). Many triazole-resistant isolates from aspergillosis patients who showed treatment failure with triazole therapy have mutations within the CYP51A protein (*31**–**35*), but this is not the only mechanism of resistance. The ECV was established to define the limits of the wild-type distribution of MICs. It does not define a breakpoint but it may help identify isolates harboring mutations. In *A. fumigatus*, 2%–20% of isolates, depending on the country, require MICs above the established ECVs for azoles (*2**–**4**,**31**,**32**,**36*). In this study, ≈5% of the isolates required an MIC greater than the established ECV for itraconazole. Isolates with no *cyp51A* mutations but which require high azole MICs are not uncommon. The mechanisms of resistance in these isolates are not precisely known but are assumed to be changes in drug transporter pumps (*37**,**38*).

Azole resistance is typically associated with *cyp51A* mutations, especially the TR_34_/L98H and TR_46_/Y121F/T289A dual mutations. A total of 26%–94% of azole-resistant isolates, especially in the Netherlands, have these mutations (*1**,**15**,**20**,**31*). However, we found no isolates harboring the TR_34_/L98H mutation in this study. One isolate was found to have an amino acid substitution at M220 and an itraconazole MIC ≥32 μg/mL. Amino acid substitutions at the M220 residue in *A. fumigatus* have been shown to confer resistance to all 3 mold-active azoles and are known to be induced by prolonged use of azoles in individual patients (*39*). Another common mutation that was discovered in isolates that required increased MICs for itraconazole is I242V. The MIC required for isolates with this mutation ranges from 1 μg/mL to 4 μg/mL. There have been no reports linking this mutation to azole resistance and a relationship between this mutation and azole resistance has not been demonstrated experimentally. The mechanism causing increased MICs for other isolates is not known, a common problem for researchers working on drug resistance in *Aspergillus* spp. (*3**,**32*).

Genetic mutations in *A. fumigatus cyp51A* are believed to be associated with exposure to azole compounds (*19**,**34**,**37**,**39*). The TR_34_/L98H and TR_46_/Y121F/T289A mutations specifically are believed to have originated from exposure to DMI fungicides in the environment (*10**,**13**,**14**,**25*). According to the US Department of Agriculture, agricultural producers in the United States use a lower tonnage of DMI fungicides than their counterparts in Europe and Asia (*26*). During 2010–2012, combined DMI fungicide use from states that provided isolates for this study was 381,018 kg. Of these states, California was the highest user (84,051 kg). According to the European Centre for Disease Prevention and Control, the United Kingdom used 271,124 kg of DMI fungicides during 2006–2009 (*8*). Therefore, the observed low rate of *cyp51A* mutation–dependent resistance, specifically the absence of TR_34_/L98H and TR_46_/Y121F/T289A mutations in this study than in studies from countries in Europe, Asia, and the Middle East, might be caused by differences in the extent of *A. fumigatus* exposure to DMI fungicides. The low rate of resistance in *A. fumigatus* shown is supported by results of other smaller studies in the United States (*2**,**40*).

There were several limitations to our study. First, isolates were collected passively. Some areas of the United States were overrepresented and others, such as the Midwest, were underrepresented. If the TR_34_/L98H mutation is found only in isolates from specific localities in the United States, it might have been missed during this surveillance. Second, all isolates were accepted. If surveillance was aimed specifically at isolates from patients who showed therapy failure, there may have been few isolates but those received might have demonstrated a greater prevalence of *cyp51A* mutations. Third, because isolates were received without personal identifiers and little meta data, there was no way of knowing whether multiple isolates came from 1 person.

In conclusion, this surveillance study indicates that the TR_34_/L98H mutant *A. fumigatus* that is now found throughout Europe has not yet emerged in the United States. Approximately 5% of *A. fumigatus* isolates from the United States required increased MICs for itraconazole, but most isolates did not have a detectable genetic mutation. In light of increasing reports of TR_34_/L98H-mediated and other *cyp51A*-mediated drug resistance in Europe and Asia, further surveillance is warranted.
